# A75 EARLY-LIFE IRON SUPPLEMENTATION SHAPES GUT MICROBIOTA AND MODULATES RECOVERY FROM GUT DYSBIOSIS

**DOI:** 10.1093/jcag/gwae059.075

**Published:** 2025-02-10

**Authors:** T Maumy, M M Santos

**Affiliations:** Centre de Recherche du Centre Hospitalier de l’Universite de Montreal, Montreal, QC, Canada; Centre de Recherche du Centre Hospitalier de l’Universite de Montreal, Montreal, QC, Canada

## Abstract

**Background:**

Iron is an essential micronutrient, but its supplementation carries significant risks to intestinal health. Excess free iron in the gut fosters pathogenic bacterial growth, disrupting the microbiota and increasing susceptibility to future intestinal stresses. Early life represents a critical window for microbiota establishment, yet little is known about the long-term effects of iron supplementation during this period. This raises concerns about how early iron exposure may impact the gut’s resilience to future stressors, such as gut dysbiosis, especially given the prevalence of intestinal stress and the widespread availability of over-the-counter iron supplements.

**Aims:**

Our study aims to investigate how early-life iron excess influences the gut microbiota and its capacity to recover from two distinct dysbiosis models:

1- Colitis-induced dysbiosis via dextran sulfate sodium (DSS)

2- Antibiotic-induced dysbiosis via a metronidazole and ciprofloxacin cocktail.

We hypothesize that early exposure to excess iron impairs microbiota recovery compared to exposure during adult life.

**Methods:**

Using C57BL/6 mice, we compared the outcomes of iron-sufficient and iron-excess diets initiated at weaning and continued into adulthood. After a 5 week exposure and an iron-sufficient period of two weeks, dysbiosis was induced, followed by an 8-week recovery phase, with frequent stool sampling for 16S rRNA sequencing to track gut microbiota composition. A bioinformatic pipeline was developed to analyze microbiota recovery and the influence of iron exposure. Parallel experiments on adult mice were conducted to explore the role of age in recovery outcomes.

**Results:**

Preliminary data indicate that the colitis model was effective, with disease severity scores confirming a stronger inflammatory response in mice exposed to excess iron during early life. Young mice in the iron-excess group showed heightened susceptibility to DSS-induced colitis compared to their iron-sufficient counterparts. Our bioinformatics workflow has been optimized for other datasets, showing reduced computational time while maintaining analytical precision.

**Conclusions:**

These findings suggest that early-life iron excess impairs gut resilience, potentially predisposing individuals to heightened responses to gut stresses like colitis or antibiotic exposure. Ongoing 16S analysis will further delineate recovery patterns, providing critical insights into how iron supplementation during infancy may influence long-term intestinal health and informing future dietary recommendations for young infants.

**Funding Agencies:**

Institut du Cancer de Montreal, Centre de recherche du CHUM, University of Montreal

